# Deceptive Triple-Negative Breast Cancer of Intermediate Grade: A Case of Rare Microglandular Adenosis-Associated Carcinoma

**DOI:** 10.7759/cureus.39531

**Published:** 2023-05-26

**Authors:** Sayed Ali I Almahari, Nisha Chandran, Reem J Maki

**Affiliations:** 1 Department of Pathology: Anatomical Pathology, Salmaniya Medical Complex, Manama, BHR; 2 Department of Radiology, Salmaniya Medical Complex, Manama, BHR

**Keywords:** benign and malignant breast lesions, microglandular adenosis associated carcinoma (mgaca), microglandular adenosis, breast cancer pathology, triple-negative carcinoma of the breast

## Abstract

Breast cancer is the most common cancer among women and the leading cause of cancer-related deaths globally. Ductal carcinoma of no special type is the most prevalent, followed by lobular carcinoma. Finding a triple-negative breast cancer of intermediate grade on core biopsies should raise the possibility of dealing with one of the rare subtypes such as microglandular adenosis (MGA)-associated carcinoma.

Here, we present a case of a 40-year-old female, who presented with bilateral breast masses, in which one of them was a high-grade carcinoma and the other turned out to be an MGA-associated carcinoma, which was misdiagnosed initially on the core biopsy as a grade II triple-negative ductal carcinoma of no special type.

Such diagnosis is challenging to pathologists, especially on small biopsies where the full morphological spectrum is not evident.

## Introduction

Breast cancer is the most prevalent cancer affecting women and the leading cause of cancer-related deaths world widely [1]. In 2020, there were 2.3 million diagnosed with breast cancer, and approximately 30% of them died because of the disease [2]. Breast cancer is the most common cancer among women in all Arab countries, and it is the leading cancer in women in the Gulf Cooperation Council (GCC). The Kingdom of Bahrain has the highest proportion (54.4%) as compared with other GCC countries [3].

There are various types of breast cancer. In a previous study conducted in The Kingdom of Bahrain, ductal carcinoma was the most common, followed by lobular carcinoma. Fifteen percent (15%) of the cases were triple-negative cancers (estrogen receptor-/progesterone receptor-/tyrosine-protein kinase erbB-2 - (Her-2 -)), of which 70% were high-grade cancers [4]. Triple-negative is a diverse group of mammary malignancies; mostly, it is composed of high-grade ductal carcinomas or metaplastic carcinomas, which are high-grade tumors [5,6].

Having cancer with lower grades and triple negativity should raise the suspicion of dealing with specific types, such as one of the salivary gland carcinomas, or some rarer tumors, like microglandular adenosis (MGA)-associated invasive carcinoma. MGA is a rare benign mammary tumor, composed of small, round single layered structures with a haphazard non-lobulocentric pattern. Despite being benign, it may be a precursor for invasive carcinoma in 25% of the cases [7].

In this case report, we present a case of a 40-year-old female with bilateral breast masses and bilateral mastectomies performed, in which the left side shows this rare entity.

## Case presentation

This is a case of a 40-year-old non-Bahraini female, a known case of hypertension and diabetes mellitus, who presented with a right breast mass for three weeks duration. There was no history of nipple discharge or any changes in the skin. The patient noticed a dramatic loss of weight over a period of a few weeks before the presentation. She has no family history of breast or ovarian carcinomas or a history of breast masses. 

On examination, a right breast upper inner quadrant hard (1 o'clock) mass measuring 3x2 cm was noted, without enlarged palpable lymph nodes in the axilla.

**Figure 1:**
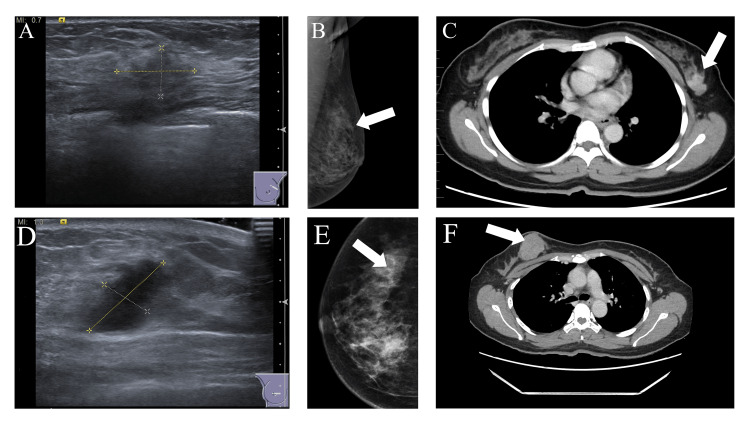
Radiology findings of both the left and right sides (A) Gray-scale ultrasound shows an irregular hypoechoic lesion at 2 o'clock on the left breast. (B) The left mediolateral oblique (MLO) mammogram shows an equal-density mass with speculated margins in the upper outer quadrant of the left breast. (C) Contrast-enhanced CT scan of the chest shows an enhancing soft tissue lesion with irregular margins at the upper outer quadrant of the left breast. (D) Gray-scale ultrasound shows a hypoechoic lesion with an irregular margin, taller than wider with surrounding architectural distortion at 1 o'clock on the right breast. (E) The right CC mammogram shows an irregular, dense, spiculated mass lesion in the right inner quadrant. (F) Contrast-enhanced CT scan of the chest shows a well-defined, heterogeneously enhancing lesion with a necrotic component associated in the upper inner quadrant of the right breast with enlarged metastatic axillary lymph nodes.

A mammogram was done for both sides, which revealed a right-side focal area of increased density with enlarged lymph nodes in the right axilla. Adding to that, a left-side mammogram detected an irregular hypoechoic lesion in the upper outer quadrant (2 o'clock) (Figure 1).

Later on, biopsies were obtained from both sides, which were diagnosed as invasive ductal carcinoma of no special type, grade III and grade II, on both the right side and the left side, respectively. The right breast carcinoma was ER/PR positive while Her-2 was negative. The left breast carcinoma was ER/PR/Her-2 negative.

A few weeks later, we received bilateral mastectomy specimens, in which the right side was a modified radical mastectomy while the left side was a simple mastectomy with sentinel lymph node biopsy.

On grossing, the right side revealed a 5cm hard white mass in the upper inner quadrant while the left side revealed a 3.5 cm hard white mass in the upper outer quadrant.

**Figure 2:**
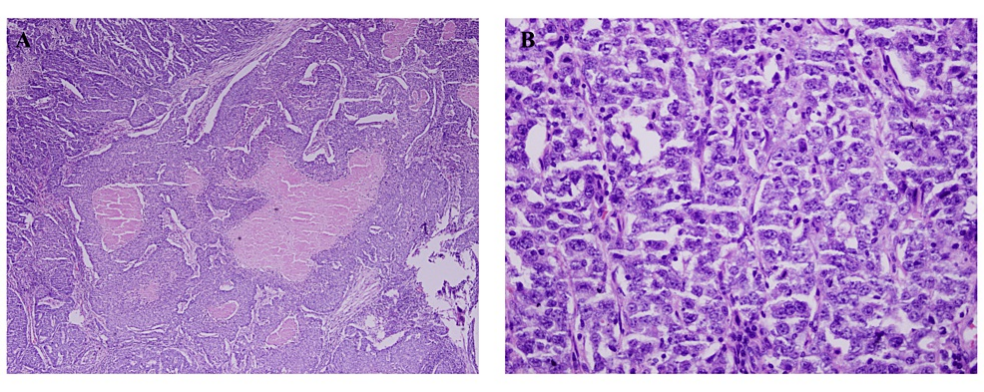
Histologic findings of the right breast (A) A low-power view of sheets of highly malignant-looking cells with extensive areas of necrosis, H&E 4x; (B) Higher-power view revealing the distinct nucleoli with brisk mitotic activity of the tumor cells, H&E 20x.

**Figure 3:**
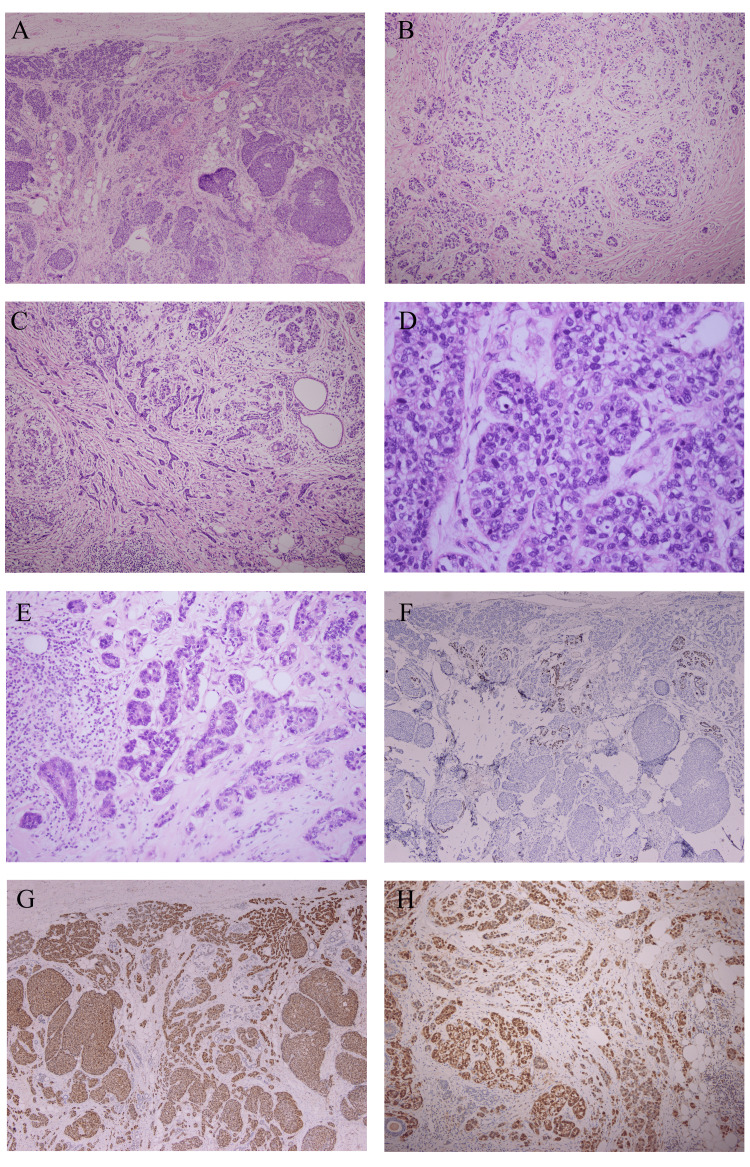
Histologic findings of the left breast (A) A low-power view of nests and tubules infiltrating in between the normal mammary parenchyma and fibro-adipose tissue, H&E 4x. (B) Chondroid metaplasia in the tumor, H&E 10x. (C) Intermediate power of infiltrative strands of tumor tubules in between the normal mammary parenchyma, H&E 20x. (D) A high-power view of the atypical MGA, revealing nests with atypical cells and frequent mitotic figures, H&E 40x. (E) Higher power of the invasive MGA, revealing strands and tubules infiltrating into adipose tissue with desmoplastic stroma in the background, H&E 40x. (F) P63 immunostaining revealing loss of myoepithelial layer, 10x. (G and H) S100 protein has strong positivity in the tumor, 10x and 20x, respectively.

Microscopically, the tumor on the right side was composed of sheets of highly malignant-looking cells with extensive areas of necrosis (Figure 2). On the other hand, the tumor on the left side showed a tumor with infiltrating mono-layered tubules with moderate atypia involving the fibro-adipose tissue and breast parenchyma. Focally, these tubules show inspissated eosinophilic secretions. Most of the areas show cells with similar morphology forming a complex architecture in the form of sheets, nests, and singly scattered cells. Foci of chondroid metaplasia and clear cell changes were seen (Figure 3).

The tubules and complex nests lacked p63 and CK5/6. They were negative for epithelial (CAM5.2 and EMA) and neuroendocrine markers (synaptophysin and chromogranin) while they were focally positive for Pan-CK. They were diffusely positive for S-100 and E-Cadherin. The tumor was negative for ER, PR, and Her-2 (Table 1).

**Table 1:** Comparison between the left and right breast cancers A comparison between both sides of breast cancer, in view of the sample, gross, histology, borders, size, axillary lymph nodes excision, background breast, presence of in-situ and invasive components, hormonal panel, and immunohistochemistry.

	Left	Right
Sample	Simple mastectomy	Modified radical mastectomy
Gross	Hard white mass	Hard white mass
Histology	Grade 2 (tubules) invasive carcinoma morphology	Grade 3 (sheets) invasive carcinoma morphology with extensive necrosis, brisk mitotic activity, and marked pleomorphism
Borders of the lesion	Irregular borders	Well-circumscribed
Size	3.5cm	5cm
Axillary lymph nodes	3 sentinel lymph nodes, all were negative for metastatic tumor	Axillary dissection was done, with 5 positive lymph nodes
Background	Benign MGA, atypical MGA, fibrocystic changes, and UDH	Fibrocystic changes and UDH
Invasiveness	Invasive component present	Invasive component present
In-situ component	Not identified	Present
Hormonal panel	ER status: Negative. 0%. (H-score: 0/300). PR status: Negative. 0%. (H-score: 0/300). Her-2 IHC status: Negative. 0/3. Proliferation index Ki-67: 40%.	ER status: positive. 40%. (H-score: 130/300). PR status: positive. 40%. (H-score: 150/300). Her-2 IHC status: Negative. 0/3. Proliferation index Ki-67: 70%
IHC	The invasive component is positive for S100 protein and E-cadherin, while negative for p63, EMA, CK5/6, CAM5.2, Synaptophysin, and Chromogranin. They are focally positive for Pan-CK.	

Initially, we thought that we are dealing with a primary salivary type carcinoma of the breast; however, with the presence of the full spectrum (benign MGA, atypical MGA, and the invasive component) and the strong S-100 protein positivity by immunohistochemistry, we concluded that this is a case of MGA with atypical changes and associated invasive carcinoma.

Unfortunately, as the patient left for her home country for further treatment and the follow-up was lost.

## Discussion

Microglandular adenosis (MGA) is an uncommon borderline neoplastic lesion, which may be associated with invasive carcinoma on rare occasions. Benign MGA is an uncommon mammary lesion occurring between the age of 28-82, exclusively in females [8]. Benign MGA may mimic invasive malignancies both grossly and microscopically, as it lacks a myoepithelial layer and it depicts an infiltrative pattern from low power [9,10]. MGA can rarely be associated with atypical growth patterns and even invasive malignancies [11].

Clinically, they may be detected as a mass lesion or as an incidental finding, like in our case [10]. From a radiological point of view, it can be found as a hyperdensity or microcalcification on mammography and an irregular hypervascular mass on ultrasonography [11,12]. In our case, the mass was detected on contrast-enhancing CT scan, as a heterogenous irregular lesion. Revision of the mammogram did not show any calcifications.

Various studies have reported associated invasive malignancy in 25-27% of MGA cases [7,13]. Usually, in the cases of malignancy, there will be a full range of benign to atypical proliferation and malignancy at the end of the spectrum [11]. The benign MGA shows small round tubules lined by a single layer of flat to cuboidal bland epithelial cells, with central eosinophilic PAS diastase-resistant secretions. The myoepithelial layer is lost, and it can be easily recognized by doing any myoepithelial staining; however, stains for the basement membranes, like collagen IV and laminin, will be preserved. On the other hand, the atypical MGA depicts gland irregularity, fusion, bridging, and budding, along with mild to moderate nuclear pleomorphism, hyperchromasia, and prominent nucleoli. Apoptotic bodies and mitoses may be present, and the epithelium is usually stratified, which was found in our case (Table 2) [10,11].

**Table 2:** Features in the differential diagnosis of MGA MGA: microglandular adenosis Source: [10]

	MGA	Atypical MGA	MGA-associated invasive carcinoma	Well-differentiated invasive ductal carcinoma
Collagen IV	Positive	Positive	Negative	Negative
S100 protein	Positive	Positive	Positive/ Negative	Negative
ER/PR	Negative	Negative	Negative	Positive (diffuse)
Myoepithelial markers	Negative	Negative	Negative	Negative
EMA	Negative	Negative	Negative	Positive
Desmoplasia	Absent	Absent	Present	Present
Cytological Atypia	Minimal	Minimal to mild	Moderate to severe	Minimal to mild
Growth Pattern	Infiltrative	Infiltrative	Infiltrative	Infiltrative
Gland Shape	Round with secretions	Variable (round to angulated, fused, complex)	Irregular	Variable (round to angulated, complex)
Atypical Cytoplasmic Snouts	Absent	Absent	Absent	Absent

The malignancy has a nesting pattern with a desmoplastic stromal reaction and a lymphocytic infiltrate. Moreover, the basement membrane is disrupted and lost [11]. A wide variety of histological appearances are reported in the literature, which includes high-grade tumors, chondromyxoid differentiations, spindle cell areas, secretory differentiation, squamous metaplasia, and basaloid features [11,14]. Features of adenoid cystic carcinoma and acinic cell carcinoma have been described [15,16].

Immunohistochemically, the whole spectrum is positive for cytokeratin and S-100 protein while negative for all the hormonal markers [17]. Ki-67 and P53 are expressed more at the malignant end of the spectrum [18]. The Ki-67 in our case was around 40% and the P53 was totally negative in the tumor cells.

In general, triple-negative mammary carcinomas have a very poor prognosis, but this is not the story of MGA-associated invasive carcinomas. Many studies suggest that such tumors' prognosis is much better [7, 19]. In one of the largest series, James et al. found that out of the 13 cases of invasive carcinoma arising from MGA, a single patient had a recurrence. In this single case, a lumpectomy was done without any further treatment [19]. 

Differentiating this entity from metaplastic and tubular carcinomas is essential, for malignant and benign MGA, respectively. First of all, having the full spectrum of MGA in the same case supports the diagnosis of MGA-associated invasive carcinoma [11]. Moreover, metaplastic carcinomas diagnosis usually requires the presence of invasive mammary carcinoma or an in-situ component in the same case, along with the support of epithelial differentiated immunohistochemically [20]. Although there are some reported cases in the literature presenting MGA-associated invasive carcinomas with in-situ components in the background; however, there was no clone relationship between the in-situ component and the invasive component in those cases [21]. Adding to that, metaplastic carcinomas are usually positive for p63, CK5/6, AE1/3, MNF116, and 34betaE12 [20]. Our case was negative for p63, CK5/6, and CK 34betaE12 while focally positive for AE1/AE3. On the other hand, tubular carcinomas can be easily confused with the benign counterparts of MGA; however, tubular carcinomas are low-grade tumors with opened lumens in a desmoplastic stroma. They are almost always positive for ER and PR while negative for Her-2 [20].

## Conclusions

We present a case of a triple-negative breast carcinoma, which was associated with MGA. Finding an intermediate-grade malignancy on a core biopsy with triple negativity should raise suspicion of rare triple-negative tumors, like the salivary counterparts or MGA-associated invasive malignancy. It is important to differentiate those tumors from ordinary triple-negative tumors, which have a much worse prognosis.
